# To Trade or Not To Trade? Criteria for Applying Cap and Trade

**DOI:** 10.1100/tsw.2001.376

**Published:** 2001-11-30

**Authors:** Stephanie Benkovic, Joseph Kruger

**Affiliations:** United States Environmental Protection Agency, Washington, DC 20460, USA

**Keywords:** cap and trade, sulfur dioxide (SO_2_), Acid Rain Program, U.S. SO_2_ emissions trading program

## Abstract

The use of emissions trading (cap and trade) is gaining worldwide recognition as an extremely effective policy tool. The U.S. Sulfur Dioxide (SO_2_) Emissions Trading Program has achieved an unprecedented level of environmental protection in a cost-effective manner. The successful results of the program have led domestic and foreign governments to consider the application of cap and trade to address other air quality issues. Certain analyses are particularly important in determining whether or not cap and trade is an appropriate policy tool. This paper offers a set of questions that can be used as criteria for determining whether or not cap and trade is the preferred policy approach to an environmental problem.

## INTRODUCTION

The first national emissions cap and trade program, the U.S. $SO_2$ Emissions Trading Program (also known as the Acid Rain Program), received worldwide attention for its environmental and economic effectiveness. The program has reduced an unprecedented 4 million tons of $SO_2$ annually from participating utility sources[1]. These emissions reductions have also led to improvements in ambient air quality and $SO_4$ deposition reduction in the northeastern U.S., where ecosystems are particularly sensitive to acidic deposition[2,3,4]. These air quality improvements and environmental and human health benefits were achieved at a fraction of the expected costs[1,5,6,7]. The cost savings in this program come from the flexibility granted to sources in determining how to achieve the needed emission reductions and the competition sparked among emission reduction strategies[8,5,9,10]. The environmental and economic success of this program has stimulated policymakers at home and abroad to consider applying cap and trade programs to address additional air pollution problems[11].

### Description of Cap and Trade

In a cap and trade program, an absolute limit (a cap) is set on total mass emissions for a group of sources for a fixed compliance period. The cap is then subdivided into allowances, each representing an authorization to emit a specific quantity of pollutant (e.g., 1 ton of $SO_2$). These allowances are allocated or auctioned by the regulating authority to the emissions sources included in the program.

During the compliance period, each source must carefully measure and report its total emissions. At the end of the compliance period, each source is required to surrender allowances to cover each ton it emitted. If a source has insufficient allowances to cover its emissions, automatic penalties are imposed.

### Recent Interest in the U.S. $SO_2$ Cap and Trade Model

The success of the U.S. $SO_2$ trading program has spurred policymakers to consider using emissions trading as part of the solution to other regional air quality problems in the U.S. A cap and trade program, known as the $NO_x$ Budget Program, was collectively established by 12 states within the Northeast (the Ozone Transport Region) to help reduce the unhealthy smog levels that pervade the area during the summer months. As a result, over 240,000 tons of $NO_x$ were reduced during the 1999 $O_3$ season, about 20% more than required. This cap and trade program for

$NO_x$ will be expanded to other states to further reduce interstate transport of $NO_x$ emissions.

Interest in cap and trade has also spread outside of the U.S. Countries using or considering cap and trade programs to control conventional air pollution include Chile, Slovakia, Poland, and China[11,12]. Cap and trade programs for greenhouse gases have been announced in Denmark and the U.K., and the European Commission has proposed a Europe-wide greenhouse gas emissions trading program[13]. Countries facing dual pressures of economic growth and environmental improvement find the cap and trade approach attractive since it can achieve significant emission reductions during times of economic growth[11].

Experience has shown that cap and trade can be a powerful policy tool when applied properly to environmental problems. However, it might not be the right tool for every air pollution situation. Several other policy options, such as emissions taxes and technology standards, can be used effectively to address pollution problems. A thorough discussion of the relative merits of these policy instruments is discussed elsewhere[14]. Similarly, although many other forms of emissions trading exist—credit trading, emissions averaging, etc.—only cap and trade is discussed in this paper.

Finally, before a decision is made on any emission reduction program, there are a number of scientific, technological, and other questions that should be answered. For example, there must be an understanding of the nature of the environmental or health problem of concern, the pathways of exposure, the location and magnitude of the emissions sources that contribute to the problem, and the levels of emission reduction necessary to address the problem. Similarly, there should be answers to technical and economic questions such as the cost, availability, and performance of control technologies. A full discussion of these questions is beyond the scope of this article. Rather, we focus our attention on whether cap and trade would be a suitable policy assuming the above-mentioned questions have been assessed.

## QUESTIONS AND CRITERIA FOR CAP AND TRADE APPLICATIONS

This section offers a framework of questions for examining the appropriateness of using cap and trade as a policy tool.

### Can the Environmental or Health Problem Be Addressed with Flexibility?

Cap and trade is premised on the notion that regulators need not direct the level or location of specific emission reductions. Instead, these programs set an overall target and let "the market" determine where the most cost-effective reductions should be made. In some cases, however, it does matter where an emission reduction is made. For example, some toxic emissions may only have local health impacts in the area immediately surrounding a facility. Allowing such a facility to buy allowances from other facilities may not fully address the risks caused by its emissions. In fact, it may make a situation worse by causing a "hotspot", i.e., an unacceptably higher accumulation of the pollutant in a specific geographic area. Such a case may necessitate controlling all facilities emitting the substance at a certain level, negating the flexibility inherent in an emissions trading program.

In general, the more a pollutant is uniformly mixed over a larger geographic area, the more appropriate it is for the use of cap and trade. For example, greenhouse gases are particularly well suited for emissions trading because their impacts upon climate change are not related to the geographic location where they are emitted. Emission reductions made in China have the same impact on atmospheric concentrations of greenhouse gases as reductions made in the U.S.

Even when the location of the emissions matters, cap and trade may be effective if the environmental goal can be met through emissions reductions in a general region. For example, a cap and trade program can reduce total loadings of a pollutant into the atmosphere, particularly if these pollutants are transported over a larger geographic region. This was the case in the U.S. with the U.S. $SO_2$ Emissions Trading Program, which is intended to reduce acid deposition in the northeastern U.S. and Canada. Similarly, cap and trade programs can address ambient air quality problems by reducing emissions transport that contributes to adverse air quality. For example, if pollution flows west to east, then it may be necessary to include emission sources west of the polluted area that may prevent areas in the east from meeting their ambient air quality standards. The $NO_X$ cap and trade programs in the northeastern U.S. have been designed to achieve large regional emissions reductions that will reduce long-range transport of $NO_X$ emissions that lead to ozone formation[15].

There are ways to assess the potential for "hotspots" and develop features to avoid them if necessary. For example, if "hot spots" are expected to arise in a particular area, restrictions can be imposed to prohibit flows of allowances into the area. Alternatively, the use of allowances may be discounted or limited within an area of concern. This may be particularly necessary for programs developed in a smaller geographic region where each source contributes more significantly to an environmental problem. It should be noted, however, that if a program requires too many restrictions on trading to avoid hotspots, it may be preferable to develop a more conventional regulatory approach to address the problem.

Just as it sometimes matters "where" an emission reduction is made, it also may matter "when" the reduction is made. Allowances are typically allocated for use in a specific compliance period. However, consideration should be given as to whether unused allowances from one compliance period can be used for compliance in future periods. This type of "temporal trading" is referred to as "banking. " Allowing banking in a cap and trade program creates additional flexibility for sources, encouraging early emission reductions, and further reducing the compliance costs.

Although the ability to bank allowances in a cap and trade program can provide significant reductions early in the program, banking can also delay the achievement of the emissions reduction target later in the program when banked allowances are used. Because banking does not delay achievement of cumulative reductions, this tradeoff does not represent an environmental concern for problems such as acid deposition and climate change. However, for problems such as ground-level $O_3$, where the environmental problem is caused by short-term episodes of high emissions, analysis should be undertaken to weigh the potential effects of banking. Experience with limits on banking in the northeastern U.S. $NO_x$ Budget Program and Southern California's RECLAIM program has shown that such limits complicated or hindered the operation of trading programs[16,17]. Therefore, these restriction types should only be adopted after careful analysis.

## Can Emissions Be Measured Accurately and Consistently?

Monitoring plays a particularly important role in a cap and trade system. Unlike many types of environmental regulation where compliance is judged by adherence to detailed technology or process specifications within a permit, cap and trade programs require a purely performance-based test for compliance. Ultimately, monitored emissions dictate how many allowances a source will have to surrender at the end of the compliance period. If one emitting facility uses a less-accurate emissions measurement method than another and consequently underestimates its actual emissions, then it could surrender fewer allowances than are necessary to cover their actual emissions. If this occurred, the environmental goal (or cap) would not be met. In contrast, monitoring for traditional command-and-control regulation, if it is required at all, is often aimed at ensuring the installation of a specified technology or an emissions rate is achieved.

Under a cap and trade system, total mass emissions (rather than just the emissions rate or concentration) must be measured from each individual unit or facility affected by the program. This complete and consistent accounting of emissions is essential to ensure that the environmental goal of the program is achieved and that the overall emissions cap is maintained. For some types of sources, total mass emissions may be difficult to measure or estimate. For example, it may be difficult to accurately account for all fugitive emissions from an industrial process, and such sources may not be amenable to a cap and trade approach.

With a cap and trade program, accuracy of measurement and consistency among sources is even more important than using consistent measurement methodology over time. Program designers aim for accuracy even if that means using an improved (but different) methodology during the compliance period than was used to establish the allocation baseline. In contrast, an emissions inventory compiled for scientific purposes, such as to model future environmental impacts of an emission, may rely on the best available existing data, even if they are inconsistent across sources.

Finally, because of the desire to ensure that each ton emitted from the stack has been accounted for, monitoring protocols for cap and trade program emphasize, not underestimate, emissions. Therefore, there is often a need for a standard methodology for substituting missing data periods. In the case of the U.S. $SO_2$ program, these missing data rules require a very conservative substitute value, e.g., a value that would result in the maximum potential emissions, to be used for any hours of missing emissions data.

## Are There Differential Marginal Costs of Abatement Across Facilities?

Cap and trade programs are most logical when emissions sources have a variety of costs for reducing emissions. These cost differences may result from the facility age, technology availability, location, fuel use, and other factors. For example, in the U.S. $SO_2$ Allowance Trading Program, there was considerable diversity in emission reduction costs because of differences in the age of power plants and the proximity to low sulfur coal supplies[18]. Where costs are different, there is "room for a deal", as high-cost sources have the incentive to buy allowances from low-cost sources. Conversely, if affected sources tend to be relatively homogenous, their marginal control costs may be approximately equal, and there is no incentive for trading. In this case, an emissions cap and trade program is unlikely to yield a significantly more cost-effective outcome than more conventional types of regulation.

## Is There an Appropriate Number of Sources?

In general, cap and trade programs should include enough sources to create an active market for allowances. If there are too few sources, there may be few trading opportunities. In addition, even if there are cost-effective trading opportunities in a program with few sources, a static market may make potential sellers reluctant to part with their excess allowances. These potential sellers may be concerned that if business conditions change and they need more allowances in the future, they will have difficulty purchasing them. They may instead horde their excess allowances even though it might not appear to be economic to do so. Finally, with fewer sources, there may be more concern that larger sources may exert market power and withhold allowances from the market to drive up prices.

There is a tradeoff, however, in that the more numerous the sources are, the more complex it becomes to establish a cap and trade program. For example, a cap and trade program that allocated allowances to every automobile driver would be difficult to implement because the administrative costs of tracking allowances and monitoring and reporting vehicle emissions would likely be substantial. Thus, the number of participating sources should be sufficient to provide for a fluid market, yet not so many that program administrators are unable to effectively manage the emissions and allowance data.

## Are There Adequate Political and Market Institutions to Enable Cap and Trade to Work?

For the *trading* part of a cap and trade program to work, a country must have some of the same institutions and incentives in place as are required for any type of market to function. These include:

- A developed system of private contracts and property rights,
- A private sector that makes business decisions based on the desire to lower costs and raise profits, and
- A government culture that will allow private businesses to make decisions about "how" to reduce emissions with a minimum of intervention.

As with all environmental programs, a cap and trade program requires adequate enforcement to ensure that environmental objectives are met. However, unlike other policy instruments, cap and trade requires credible enforcement for its cost-saving objectives to be met. For an emissions market to develop, there must be confidence that emissions will be correctly measured and reported, that compliance will be verified, and, if there is noncompliance, that a significant cost will be assessed. Thus, cap and trade programs will have greatest success in countries where rule of law is respected and enforcement is consistent, impartial, transparent, and politically independent. Participants should clearly understand from the beginning how the program works and how compliance will be measured and enforced. The credibility of an emissions trading market will diminish significantly if firms believe that rules are unfair, arbitrary, or unpredictable.

Another important consideration is whether the relevant government entity has sufficient jurisdiction over the geographic area where the cap and trade program would be implemented. In many countries, environmental programs are implemented by regional or local authorities who must follow national policies, but are given considerable autonomy in implementation. For example, China's provincial Environmental Protection Boards (EPBs) have the main responsibility for running air quality and other environmental programs. Similarly, in Slovakia there are 79 local districts that implement environmental and other programs. To the extent that cap and trade programs cover a geographic region with more than one local authority, there must be some consistency in key design elements of the program. For example, in multistate $NO_x$ trading programs in the northeastern U.S., there are common requirements for emissions measurement and reporting, common standards for enforcement, and common penalties. Other design elements, such as allocation methodologies, might be left to the provinces or municipalities since they have no environmental impact. Allowing different provinces or municipalities to have different allocation schemes may have competitive impacts, however, such as favoring firms within an industrial sector in one region of a country over another.

Finally, even if a country does not yet have all of the attributes described above, it still may be beneficial to develop the infrastructure necessary for a cap and trade program in advance of more comprehensive economic and political changes[17]. As centrally planned economies transition to become more market-oriented, they may also transform their environmental programs to become more effective. Even if conditions are not yet ripe for trading, the structure of a cap and trade program may improve environmental performance. In particular, the emphasis on careful mass-based emissions measurement and accounting may improve the environmental accountability of sources. For example, recent experiments in Slovakia and Chile have indicated that the allocation process associated with cap and trade has served as an incentive for more complete and accurate emissions inventories[12,13].

## CONCLUSIONS

Beginning in 1995, the U.S. Acid Rain Program was the first national cap and trade program for controlling air pollution in the world. The results of the program have been exceptional both in terms of the magnitude of emission reductions and the minimization of compliance costs among participating sources. Today, cap and trade programs are being seriously considered around the world. As described in this paper, there are many advantages to using cap and trade as a policy tool. However, it should not be applied to every environmental problem. A thorough analysis of the key questions examined in this article can help to ensure that a cap and trade program will deliver intended environmental and economic benefits.

## ACKNOWLEDGEMENTS

The authors would like to thank all their colleagues in the EPA's Clean Air Markets Division, whose input was brought to bear in many aspects of this paper. In particular, the authors thank Jennifer Macedonia for her contributions on the discussion of emissions measurement. In addition, the authors thank A. Denny Ellerman, Richard Morgenstern, and Noreen Clancy for their insights and input. The views expressed here are solely the authors' and do not necessarily reflect the policies of the EPA.

| | | |
|---|---|---|
| Received: | August | 21, 2001 |
| Revised: | November | 9, 2001 |
| Accepted: | November | 9, 2001 |
| Published: | November | 30, 2001 |
